# From Framework to Toolkit: Practical Guidance for Using the PrOcess Evaluation of Trials In Critical care (POETIC) Framework

**DOI:** 10.1097/CCE.0000000000001503

**Published:** 2026-10-27

**Authors:** Lydia M. Emerson, Daniel F. McAuley, Mike Clarke, Bronagh Blackwood, Gosha Colquhoun

**Affiliations:** 1 Department of Health and Community Sciences, The Medical School, University of Exeter, Exeter, United Kingdom.; 2 Wellcome Wolfson Institute for Experimental Medicine, School of Medicine, Dentistry and Biomedical Sciences, Queen’s University Belfast, Belfast, United Kingdom.; 3 Centre for Public Health, School of Medicine, Dentistry and Biomedical Sciences, Queen’s University Belfast, Belfast, United Kingdom.; 4 School of Health and Social Care, Edinburgh Napier University, Edinburgh, United Kingdom.; 5 Division of Research Methodology, Wroclaw Medical University, Wroclaw, Poland.

**Keywords:** complex intervention, critical care, health services research, process evaluation, randomized controlled trial

## Abstract

**Background:**

The PrOcess Evaluation of Trials In Critical care (POETIC) framework was developed to support systematic evaluation of Context, Fidelity, Dose, Reach, and an integrative assessment of Quality of Delivery (QoD). Its practical use, however, requires explicit guidance on selecting measures, constructing QoD, assessing changing context, and governing prospective use.

**Methods:**

We undertook a retrospective comparative synthesis of process evaluations embedded in four randomized U.K. critical care trials: Biomarker-guided antibiotic stewardship in suspected ventilator-associated pneumonia (VAPrapid-2), Provision of Psychological Support to People in Intensive Care (POPPI), Sedation AND Weaning In CHildren (SANDWICH), and Alpha-2 agonists for sedation to produce Better outcomes from critical illness (A2B). We selected them because they informed POETIC’s development or application and provided sufficiently detailed quantitative and qualitative evidence. We mapped the reports, protocols, and process evaluation findings to the five dimensions; compared recurring and contradictory patterns, quantitative-qualitative agreement, measurement and interpretation problems, and mechanism-specific limitations; and translated our learning into guidance for applying the framework.

**RESULTS:**

The relevance and measurement of POETIC dimensions varied according to how each intervention was expected to work. Contextual influences changed over time and differed between sites. Combined assessments of QoD required explicit decisions about component selection, weighting, thresholds, aggregation, and missing data. VAPrapid-2 showed why a separate Dose measure and a QoD composite were not meaningful for a one-off diagnostic recommendation; POPPI used a weighted site composite; SANDWICH summarized delivery of four intervention components, staff training, and patient Reach; and A2B combined four equally weighted indicators in a 0–12 site score. This cross-trial learning informed practical guidance on selecting measures, assessing context over time, integrating quantitative and qualitative findings, constructing QoD, and governing prospective use.

**Conclusions:**

POETIC is best used as a mechanism-aligned, mixed-method scaffold rather than a requirement to measure every dimension or calculate a universal composite. Prospective use is feasible when outputs, recipients, review times, permitted actions, and safeguards are prespecified. The framework and accompanying toolkit support reproducible assessment while protecting trial integrity.

**KEY POINTS**

**Question:** How can the PrOcess Evaluation of Trials In Critical care (POETIC) framework be translated from a conceptual model into a tool for a reproducible and responsibly governed process evaluation?

**Findings:** Cross-trial synthesis showed that context must be assessed longitudinally, delivery measures must follow the intervention mechanism, and Quality of Delivery should only be constructed when its components, thresholds, weighting, aggregation, and missing-data rules can be justified in advance. Among the four applications, the Alpha-2 agonists for sedation to produce Better outcomes from critical illness (A2B) trial used prospective process evaluation findings to develop general study implementation materials and inform later sampling, illustrating why such use requires explicit governance.

**Meaning:** POETIC can strengthen interpretation of whether an intervention was meaningfully tested, but prospective feedback must be separated from ungoverned performance management or intervention adaptation.

Process evaluation is an established component of trials of complex interventions (1, 2). It can determine whether an intervention was delivered as intended, identify mechanisms of impact, and explain why similar sites achieved different results. This matters in positive, neutral, and inconclusive trials because the delivery and contextual conditions associated with benefit inform replication, guidelines, and wider implementation (1, 3). Establishing whether an intervention was meaningfully tested is also a scientific and an ethical responsibility: without evidence about delivery and context, an inconclusive finding may be wrongly attributed to the intervention rather than inadequate delivery, reducing the value of participants’ contributions (1, 3).

Critical care makes this need particularly acute because intervention delivery occurs in high-acuity, time-pressured systems shaped by staffing, workflow, professional roles, usual practice, risk perceptions, and clinical judgment (4–6). A protocol may specify what should be done, but the relevant question is often whether it could be done, for whom, under which conditions, and consistently enough to test effect. Process evaluations nevertheless vary in their treatment of context and theory, and operational detail is often insufficient for replication (7, 8).

The PrOcess Evaluation of Trials In Critical care (POETIC) framework was developed to address this gap (9). The original framework links an intervention logic model to five dimensions: Context, Fidelity, Dose, Reach, and Quality of Delivery (QoD; **Fig. 1**). Context comprises organizational and participant-related characteristics grouped within Unit Culture, Organizational Structure, Resources, Usual Practice, and Attitudes and Perceptions. Fidelity asks whether the intervention’s core functions or components were delivered as intended. Dose describes how much of the intervention was delivered and received. Reach describes the extent to which the intended population was exposed to or engaged with the intervention. QoD is an optional integrative assessment of Fidelity, Dose, and Reach rather than an additional independent dimension (9, 10). POETIC distinguishes intervention delivery from trial processes such as recruitment and data collection and proposes assessment before, during, and at the end of a trial.

**Figure 1.**
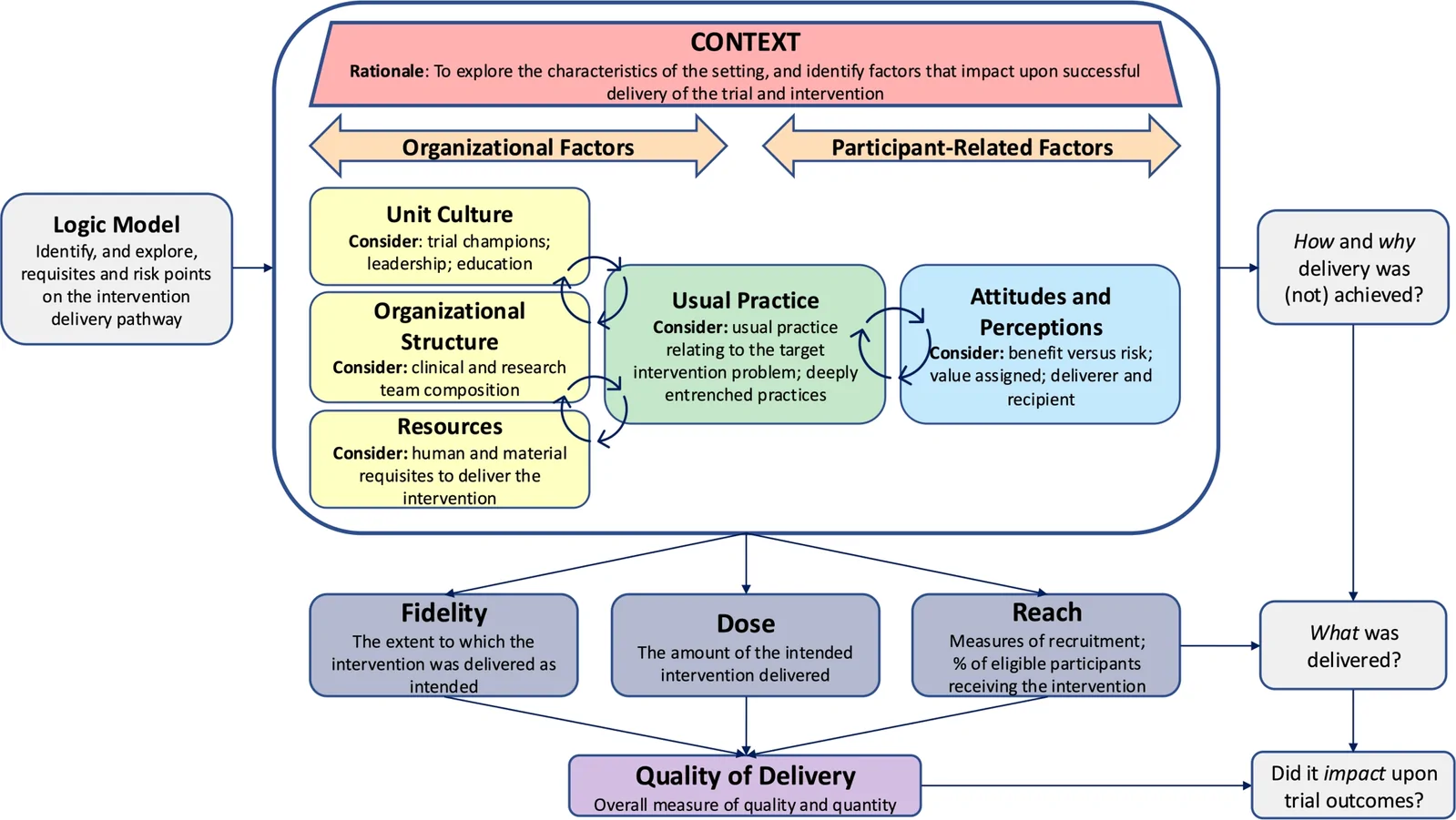
The PrOcess Evaluation of Trials In Critical care (POETIC) framework. The intervention logic model guides selection of Context, Fidelity, Dose, and Reach measures. Quality of Delivery is an optional integrative assessment of Fidelity, Dose, and Reach. Trial-process measures should be distinguished from intervention-delivery measures. Reproduced from Emerson et al (9).

The first POETIC article described the framework’s conceptual development (9). This article has two linked aims: 1) to explain what was learned from applying POETIC across four process evaluations; and 2) to translate that learning into practical guidance that trial teams can use to design, analyze, govern, and report a POETIC-informed evaluation. The article therefore provides a practical toolkit covering Context specification, QoD construction, applicability, mixed-method integration, and safeguards for prospective use.

## MATERIALS AND METHODS

### Design and Scope

We retrospectively compared process evaluations prospectively embedded in four randomized trials in U.K. ICUs: Biomarker-guided antibiotic stewardship in suspected ventilator-associated pneumonia (VAPrapid-2) (11), Provision of Psychological Support to People in Intensive Care (POPPI) (12), Sedation AND Weaning In CHildren (SANDWICH) (13), and Alpha-2 agonists for sedation to produce Better outcomes from critical illness (A2B) (14, 15). We included them because their completed evaluations used or informed POETIC dimensions, reported detailed quantitative and qualitative findings, and represented different intervention mechanisms. VAPrapid-2 and POPPI informed the framework’s development; SANDWICH and A2B were later applications before POETIC was published (9). The trials were not prospectively planned as a staged POETIC program; this cross-trial comparison was undertaken after each evaluation had been completed. We compared how the existing POETIC dimensions were applied, not intervention effectiveness.

### Sources and Trial-Specific Analytic Foundations

Sources comprised trial protocols and logic models, published main-trial reports, published process evaluation reports, quantitative routine trial data, and qualitative findings. We did not reanalyze individual-level primary data for this article. Instead, we extracted how Context, Fidelity, Dose, Reach, and integrative delivery assessments were defined; the timing and source of each measure; how qualitative and quantitative findings were combined; whether information was used during or only after trial conduct; and what operational limitation or learning arose.

The underlying qualitative methods were reported in the contributing studies. They included semi-structured interviews, site comparisons, and content analysis and matrix-based framework approaches combining deductive categories derived from intervention logic models with inductive examination of unallocated or discrepant data (16, 17). VAPrapid-2 used three phases of semi-structured interviews. Early field notes were analyzed using content analysis, and later interview transcripts were analyzed using a seven-step framework method. A second researcher checked the analysis, and a maximum-variation matrix supported comparison within and between sites (11, 18). POPPI used phased data collection, content analysis during the trial, and a seven-stage framework approach in the final phase (12, 19). SANDWICH used a framework-based mixed-method analysis with prespecified intervention components and inductive thematic content analysis; 15% of qualitative material was independently analyzed (13, 20). A2B used a seven-step framework approach, second-researcher review of the coding framework, iterative examination of unallocated data, and team consensus before mixed-method synthesis (14, 15).

L.E. contributed to three of the four applications of POETIC (VAPrapid-2, POPPI, and A2B), while the wider clinical, trial, statistical, and qualitative teams differed by study. This overlap supported continuity but also created a risk of confirmation bias, considered below as a limitation.

### Cross-Trial Extraction and Translation into Guidance

L.E. completed the initial cross-trial mapping using a common matrix organized by trial, source, POETIC dimension, timing, data type, analytic use, and practical learning. B.B. checked the mappings against the source reports. The research team then reviewed the cross-trial comparisons, considered alternative explanations, and agreed on the operational guidance by consensus. We included an observation in the guidance when:

1. it recurred across trials, sites, or phases;
2. quantitative and qualitative findings supported the same explanation;
3. it revealed a practical problem affecting measurement or interpretation; or
4. it showed that a dimension or composite would not be useful for a particular intervention mechanism.

Contradictory findings were retained to show when the guidance might not apply. Given the differences between the contributing evaluations, we did not use a numerical evidence threshold or claim thematic saturation across studies. The five POETIC framework dimensions were not revised. We used the cross-trial learning to clarify how trial teams can select measures, plan timing, interpret findings, and govern prospective use.

### Mixed-Method Synthesis

Within each trial, quantitative data described the extent and distribution of delivery, whereas qualitative data explained how and why delivery varied. Across trials, we examined whether the data sources agreed (convergence), added different parts of the explanation (complementarity), or suggested different interpretations (dissonance). We included recurring contextual factors in the recommended minimum dataset only when they were supported by a reported example or delivery pattern. Factors relevant to only one site or intervention were placed in optional modules. We distinguished changes within a site over time from differences between sites at the same time point.

### Independence from Outcomes and Use During Trial Conduct

Process evaluation data collection and analyses were completed without knowledge of primary trial results (11, 15, 19, 20). A2B explicitly reported that data collection and qualitative analyses were completed before the primary results were known (15). The other reports do not consistently distinguish results being unavailable from active blinding, so we do not claim that all four trials used the same safeguard. This separation protected interpretation but did not prevent process evaluation findings from influencing trial conduct. In A2B, Phase 1 findings informed general implementation materials and topics explored in Phase 2 (15). Their content, recipients, and timing were insufficiently reported to exclude an influence on intervention delivery or fidelity. Randomized treatments, comparators, eligibility criteria, endpoints, and outcome analysis were unchanged. This informed the governance model below: feedback that could affect delivery or affect randomized groups differently should be prespecified and governed.

### Ethics Approval and Consent to Participate

This study was a methodological synthesis of previously published process evaluations and did not involve human participants, identifiable personal data, or reanalysis of individual-level participant data. In accordance with Queen’s University Belfast Research Ethics Committee policy, this activity did not constitute human subjects research and was therefore exempt from ethics review. Consent to participate was not required.

## RESULTS

### What We Learned From Four Trial Applications

**Table 1** restates the original POETIC dimensions (9) and shows how learning from the four applications translates into practical guidance. The five framework dimensions are unchanged.

**TABLE 1.** PrOcess Evaluation of Trials In Critical care (POETIC) Dimensions and Practical Guidance

Dimension or Application Principle	Original Analytic Purpose	Practical Guidance From the Four Applications
Context	Explain organizational and participant-related influences on delivery	Use a minimum longitudinal core, distinguish temporal from between-site variation, and add intervention-specific modules
Fidelity	Assess delivery of core functions/components as intended	Define core versus adaptable components from the logic model and assess delivery only on eligible occasions when the component could have been provided
Dose	Assess amount delivered and received	Use only where exposure has meaningful quantity, intensity, duration, or frequency; do not force Dose for binary or one-off mechanisms
Reach	Assess exposure or engagement of the intended population	Separate patient, staff, site, and trial-process Reach; prespecify denominator and eligibility windows
Quality of Delivery	Integrate Fidelity, Dose, and Reach	Treat as optional; prespecify components, thresholds, weighting, aggregation, missing-data rules, and intended analysis; retain component results alongside any composite
Timing/use	Assess before, during, and after trial delivery	Declare the intended mode: end-of-trial explanation, prospective monitoring, or governed interpretive use, and separate interpretation from modification

### VAPrapid-2: Mechanism-Sensitive Measurement

VAPrapid-2 evaluated biomarker-guided antibiotic prescribing for suspected ventilator-associated pneumonia. A recommendation was available to inform a time-pressured clinical decision, but use depended on clinicians’ trust in the biomarker, tolerance of diagnostic uncertainty, and perceived consequences of undertreatment. Fidelity could be represented by concordance between the recommendation and prescribing decision. By contrast, Dose did not have an interpretable repeated-exposure meaning, and a forced Fidelity-Dose-Reach composite would have obscured rather than clarified delivery (11).

Qualitative data collected before, during, and late in the trial explained why recommendations were accepted or overridden and informed purposive selection of later sites. The practical lesson was not to remove Dose from POETIC, but to use it only when the intervention involves a meaningful amount, frequency, intensity, or duration of exposure. For a one-off diagnostic recommendation, Fidelity and Reach were more informative than a forced Dose measure or composite (11, 18).

### POPPI: Mixed-Method and Differentially Weighted Integration

POPPI evaluated a nurse-led psychological intervention designed to reduce posttraumatic stress disorder symptom severity after critical illness. It comprised creating a therapeutic environment, stress-support sessions, and a relaxation and recovery program. Repeatable components supported measurement of Fidelity, Dose (patient exposure), and staff and patient Reach. Qualitative Fidelity for core elements was scored from 0 (low adherence) to 3 (full adherence). Routine trial data included staff training uptake, time to achieving an 80% training target, the proportion of patients receiving two or more stress-support sessions, the proportion of highly stressed patients receiving no sessions, use of the electronic tablet program, and receipt of take-home materials (12, 19).

Investigators scored qualitative adherence criteria from 0 to 3 and combined them within four intervention components. They distributed 15 weighting points across the components to reflect their perceived importance, producing a weighted composite with a maximum of 135. Sites were ranked and divided into lowest-, moderate-, and highest-adherence tertiles according to the prespecified analysis plan. Two researchers cross-checked the qualitative-to-numeric scoring and resolved disagreements by consensus (19).

### SANDWICH: Prospective Component and Site Assessment

SANDWICH evaluated a coordinated pediatric sedation and ventilator-liberation pathway in a stepped-wedge cluster randomized trial in pediatric ICUs. Delivery assessment covered three separate elements: adherence to four core intervention components; staff training completion by 8 weeks; and patient Reach, calculated as screened admissions divided by invasively mechanically ventilated admissions. Component adherence was captured daily. Average adherence proportions were calculated for each unit, ranked, and divided into tertiles for a prespecified subgroup analysis (13, 20).

The qualitative analysis showed why a high training percentage did not necessarily mean that the intervention was delivered well at the bedside. Multidisciplinary working, professional roles, routines, staffing, and opportunities to provide each component remained influential. Training therefore showed whether staff had been reached and given the basic capability to deliver the intervention; it did not show whether the intervention was delivered well at the bedside. Each repeated component also needed to be assessed only on occasions when it could appropriately have been provided (20).

### A2B: Explicit Equal Weighting and Prospective Use

A2B compared dexmedetomidine- or clonidine-based sedation with propofol-based usual sedation across 38 adult ICUs (14, 15). Four site-level delivery indicators were prespecified:

- Fidelity: percentage of patients receiving the correct randomized treatment every day during the first 4 days;
- Fidelity: percentage of patients reaching a Richmond Agitation-Sedation Scale score of –2 or lighter at any point during the first 4 days;
- Dose: percentage of patients with at least one Richmond Agitation-Sedation Scale assessment per day during the first 4 days; and
- Reach: percentage of eligible patients proceeding to randomization.

Each indicator was scored 0 for less than 40%, 1 for 40–59%, 2 for 60–79%, and 3 for 80% or more. The rationale for these historical cut points was not explicitly reported in the original process evaluation documentation. The four indicators were equally weighted and summed to a site score from 0 to 12. In the observed data, sites scored 6–12 and were categorized as low (6 or 7), moderate (8 or 9), or high (10–12) adherence. Planned measurement of staff Reach using online training completion was not feasible because staff did not consistently complete the online training; the completion records therefore could not show how many staff had been reached. The historical thresholds and categories were trial-specific descriptive constructs, not validated or transferable cut points. Missing staff Reach data showed why feasibility and missingness should be assessed before relying on a composite (15).

Quantitative delivery data guided interpretation of between-site variation; purposive qualitative sampling considered recruitment, usual sedation-target practice, research-nurse resource, and unit size. Mid-trial findings informed general study implementation materials and later qualitative inquiry. They did not alter the randomized treatments, comparators, outcomes, or statistical analysis. A2B thus supports prospective interpretive use, but not an unrestricted claim that process evaluation feedback is methodologically neutral (15). **Table 2** compares how integrative delivery assessments were constructed and used across the four trials.

**TABLE 2.** Construction and Use of Integrative Delivery Assessments Across Trials

Trial	Intervention Type	Components/Data	Scaling and Weighting	Site Summary and Role	Limitation/Learning
Biomarker-guided antibiotic stewardship in suspected ventilator-associated pneumonia (VAPrapid-2)	Diagnostic/judgment-based	Recommendation-prescribing concordance plus interviews	No composite	Fidelity interpreted with Context	Dose not meaningful; Quality of Delivery not forced
Provision of Psychological Support to People in Intensive Care (POPPI)	Multicomponent, repeatable	Mixed-method Fidelity scores; training, sessions, patient and materials measures	Qualitative criteria scored 0–3; 15 weighting points distributed across components; weighted maximum 135	Ranked site composite; adherence tertiles; planned subgroup analysis	Scoring narrative data and weighting require transparent consensus; missing-data rule was not reported
Sedation AND Weaning In CHildren (SANDWICH)	Protocolized daily bundle	Four component proportions, staff training, eligible-patient Reach	Component proportions; no single universal unit scale	Mean adherence ranked into tertiles; prespecified subgroup analysis	Training did not guarantee component delivery; opportunity denominators mattered
Alpha-2 agonists for sedation to produce Better outcomes from critical illness (A2B)	Continuous clinician-titrated therapy	Two Fidelity, one Dose, one patient-Reach indicator	Each 0–3; equal weighting; total 0–12	Low 6–7, moderate 8–9, high 10–12 in observed sites	Staff Reach unavailable; prospective use requires governance

### Cross-Trial Patterns

Context was both temporally dynamic and different between sites. Workforce disruption, staffing experience, workload, concurrent studies, service pressures, and research support changed during trials; unit culture, usual practice, leadership, role boundaries, and risk perceptions also differed at the same timepoint across sites. Treating context as a one-time descriptive paragraph would have missed both forms of variation (11, 15, 18–20).

Attitudes and Perceptions repeatedly mediated delivery. In VAPrapid-2, risk perceptions and trust in diagnostic evidence shaped prescribing. In POPPI, confidence and perceived legitimacy of a psychological-support role influenced whether trained staff delivered sessions. In SANDWICH, professional roles and multidisciplinary norms shaped ventilator-liberation components. In A2B, clinician preference, equipoise, safety concerns, usual sedation practice, and staff capability influenced treatment and sedation targets (11, 15, 18–20). These findings accord with wider evidence that context, professional behavior, and theory use are important but frequently under-specified in trial process evaluations (4–8).

Across trials that measured training, high staff completion rates did not guarantee consistent delivery. Delivery could still be limited by staffing, role clarity, workflow, perceived risk, local norms, or a lack of suitable opportunities. A low component score could also reflect occasions when clinical circumstances meant that the component could not appropriately be delivered. For repeated components, the denominator should therefore include only eligible occasions when delivery was possible, and qualitative findings should explain important exceptions (15, 19, 20).

## OPERATIONAL GUIDANCE FOR APPLYING POETIC

### Start With the Intervention Logic Model and Intended Use

Before selecting data, the trial team should specify the core mechanisms through which the intervention is expected to affect outcomes, the components that are required to activate those mechanisms, and which components can be adapted without undermining the mechanism. Each dimension should answer a defined interpretive question. Trial teams should also declare whether the process evaluation is intended for: 1) end-of-trial explanation; 2) prospective monitoring with analysis completed before outcome unblinding; or 3) governed interpretive use during trial conduct. The third mode carries the greatest risk of changing behavior and requires the governance safeguards described below.

Not every POETIC dimension must yield a numeric measure. The typology in **Supplemental Table 1** (https://links.lww.com/CCX/B697) is a selection guide, not a taxonomy into which every intervention must fit.

### Operationalize Context as a Longitudinal Minimum Dataset

The Context dataset recommended in **Table 3** should be adapted to the intervention logic model and assessed at baseline, at prespecified intervals, after major triggers, and at a defined final point. The protocol should define these timepoints and state whether assessment will precede availability of comparative results. The final point may be the end of intervention delivery at a site or of the final delivery phase; it need not await completion of all participant follow-up. Intervals should reflect how quickly factors change: staffing and workload may require monthly or quarterly summaries, whereas organizational and usual-practice measures may be repeated at phase transitions or annually. Triggered assessment may follow service reconfiguration, a staffing crisis, a new guideline, a competing trial, a supply interruption, an outbreak-related surge in eligible patients, or other pressures that reduce delivery capacity.

**TABLE 3.** Recommended Minimum Context Dataset and Optional Modules

Core Domain	Minimum Variables/Questions	Suggested Timing/Data Source	Optional Module Examples
Unit culture	Norms for protocol use; learning/psychological safety; multidisciplinary working; tolerance of uncertainty	Baseline and phase/end interviews; brief repeated staff survey	Psychological support role confidence; willingness to challenge sedation practice
Organizational structure	Unit type/size; decision authority; role clarity; handover and multidisciplinary routines	Baseline profile; repeat after structural change	Stewardship approval pathways; ventilator-weaning responsibilities
Resources	Staffing level, skill mix and turnover; workload/surge; research support; equipment/supply availability	Monthly/quarterly routine metrics plus event log	Research-nurse capacity; medication or diagnostic-kit availability
Usual practice	Baseline care and distance from intervention; competing protocols/studies; relevant practice drift	Pretrial audit; repeated sample at phase transitions/end	Sedation targets and drug use; antimicrobial practice; baseline psychological support
Attitudes and perceptions	Acceptability, confidence, risk/safety concerns, equipoise, perceived burden and legitimacy	Baseline exploratory work; purposive mid-/end-trial interviews or survey	Biomarker trust; concern about light sedation; role legitimacy
External/temporal events	Policy/guideline changes; outbreaks; service reconfiguration; major workforce disruption	Prospective dated event log; triggered qualitative follow-up	Pandemic surge; publication of an influential external trial

### Construct QoD Only When it Improves Interpretation

QoD is optional. Before creating a combined score, the trial team should agree:

1. which measures will be included;
2. the eligible patients or occasions used as the denominator for each measure;
3. the cut points and why they were chosen;
4. whether components will count equally and, if not, why;
5. whether the result will remain a numeric score, be grouped into categories, or both;
6. what will happen if information is missing or very few patients contribute; and
7. how the overall score and its individual components will be reported and used.

We recommend agreeing these decisions before comparative trial results are examined. First report the completeness of each component. If an essential component is missing, report the combined score as “not classifiable.” Recalculate a total from available components, or fill in a missing value, only when a justified method was specified before trial results were known. Always report individual components alongside the overall score.

### Integrate Quantitative and Qualitative Data

Quantitative data should identify where, when, and to what extent delivery varies. Qualitative data should explain mechanisms, perceived consequences, and exceptions. Sampling should therefore be informed by the logic model and, where appropriate, by a maximum-variation matrix or prespecified triggers in delivery and Context data rather than convenience alone.

Within dimensions, analysis can combine descriptive statistics with framework analysis. Across dimensions, a site-by-time joint display should compare Fidelity, Dose, Reach, and any QoD summary with Context. Convergence strengthens an explanation; complementarity can reveal mechanism; and dissonance should prompt rechecking of denominators, timing, measurement validity, and alternative interpretations.

Qualitative findings should be converted to a score only when the team has defined a clear scoring guide, at least two researchers have checked the scoring, and the method for resolving disagreements has been agreed. Unless their measurement properties have been formally tested, these scores should be described as analytic classifications (categories created for the analysis) rather than as validated quantitative measures.

### Govern Prospective and During-Trial Use

The governance model separates data collection, outcome-blinded synthesis, review by agreed recipients, and permitted interpretive use (**Fig. 2**); **Table 4** translates this model into action boundaries and minimum safeguards. Feedback-loop methods can support timely learning (21), but exact recipients should be specified in a process evaluation charter. As a default, the process evaluation team may receive site-level delivery and Context data while remaining blinded to comparative outcomes. A designated trial-management or oversight group may receive aggregate or blinded summaries at fixed intervals.

**TABLE 4.** Governance Boundary for Prospective PrOcess Evaluation of Trials In Critical care (POETIC) Use

May be permitted when prespecified	Requires formal review/amendment or remains prohibited
Verify completeness and resolve data-definition errors	Access comparative outcome data outside authorized groups
Describe Context and delivery using fixed metrics	Change Quality of Delivery components, thresholds, or weights in response to emerging results
Trigger prespecified maximum-variation or explanatory sampling	Rank or compare named sites for performance management
Document implementation barriers and external events	Prompt additional intervention training or remedial delivery feedback unless this is a prespecified, balanced component of trial conduct
Provide aggregate/blinded interpretive summaries to named oversight groups	Modify intervention content, dose, comparator, eligibility, endpoints, or analysis without formal governance and amendment
Escalate safety, misconduct, or data-integrity concerns through existing pathways	Use process findings selectively in a way that could differentially affect trial arms or contaminate controls

**Figure 2.**
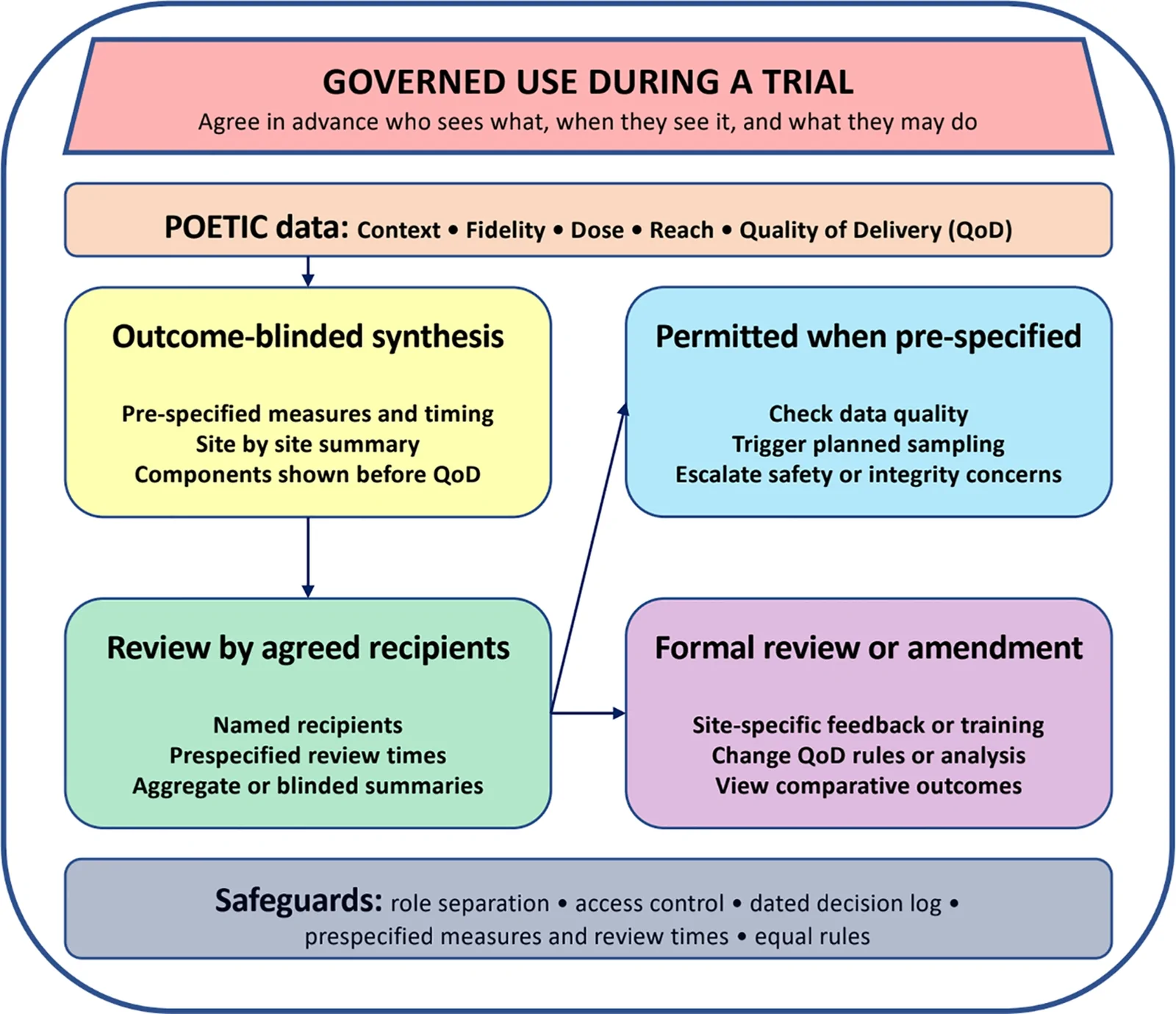
Governed data flow for prospective and during-trial use of PrOcess Evaluation of Trials In Critical care (POETIC). QoD = Quality of Delivery.

Minimum safeguards include keeping the process evaluation team unaware of comparative trial results and separating roles where feasible; limiting access to the data; using fixed measures and reporting intervals; maintaining a dated decision log; providing aggregate or blinded reports; applying the same rules across sites and randomized groups; and defining explicit escalation pathways. Reports should state what action, if any, is authorized. If feedback is intended to improve delivery, it becomes part of the intervention and should be described, standardized, monitored, and incorporated into the trial’s definition of the treatment effect.

## DISCUSSION

### Principal Findings

This synthesis provides four practical lessons without changing POETIC’s five dimensions. First, POETIC is not a universal checklist requiring every dimension to be quantified: measures should follow the intervention mechanism, and absence of a meaningful Dose or QoD may reflect valid tailoring. Second, Context should be measured over time and across sites to distinguish temporal from between-site variation. Third, QoD is an optional, prespecified summary requiring transparent choices about indicators, thresholds, weights, aggregation, and missingness. Fourth, using process evaluation findings during a trial may influence conduct: who sees information, when, and what they may do with it must be governed.

The four applications retained POETIC’s conceptual structure while tailoring its operational use. VAPrapid-2 showed the importance of mechanism-sensitive measurement for clinician judgment (11, 18). POPPI showed how qualitative and quantitative data can be integrated and the judgments involved in scoring and weighting (12, 19). SANDWICH showed prospectively defined, opportunity-based component assessment and the limitations of treating training as delivery (13, 20). A2B used explicit site scoring and showed how early- and mid-trial findings could inform implementation materials and later inquiry, and why such use requires explicit governance (14, 15).

### Relationship to Wider Evidence

The prominence of workflow, staffing, professional roles, usual practice, and perceptions is not unique to this work. It aligns with evidence on behavior change in intensive care (4–6). It also aligns with Medical Research Council guidance emphasizing context and mechanisms (1, 3), and reviews showing inconsistent theory and context specification in trial process evaluations (7, 8). The contribution of the present work is therefore not a claim to have discovered these influences, but to convert them into a critical-care-specific minimum dataset, timing strategy, and decision rules linked to delivery measurement.

POETIC complements rather than replaces broader implementation frameworks. The Reach Effectiveness Adoption Implementation Maintenance (RE-AIM) framework emphasizes population reach and maintenance (22). Cluster-trial process evaluation guidance emphasizes design and interpretation across levels (23). POETIC provides a narrower scaffold for determining whether an intervention was delivered as intended within a critical care trial and how context shaped that delivery.

### Clinical and Research Implications

Delivery interpretation can affect clinical conclusions. A neutral result with high mechanism-relevant delivery provides stronger evidence against the intervention as tested than one with poor Fidelity, implausible Dose, or low Reach. A beneficial result concentrated in a particular context or delivery profile identifies conditions needed to replicate benefit. These distinctions can shape guideline recommendations and whether investment should prioritize efficacy research, delivery redesign, or implementation support. Transparent prospective safeguards also support the scientific validity and social value expected of ethical research (24).

The four trials in this study, and the applications of POETIC within them, have direct clinical relevance. In antimicrobial stewardship, understanding why clinicians override diagnostic recommendations distinguishes assay limitations from risk perception and decision pathways (11, 18). In sedation and ventilator liberation, component-level delivery and context help determine whether protocol effects are limited by titration practice, role boundaries, staffing, or treatment mechanism (13–15, 20). In psychological support, high training uptake without patient exposure warns against assuming that workforce preparation alone translates into care (12, 19). POETIC does not prove that improving these delivery factors improves patient outcomes, but it makes the interpretation and decisions about future research more defensible.

### Strengths and Limitations

This synthesis is grounded in four large, pragmatic trials spanning adult and pediatric ICUs, using individual and cluster randomization, and testing judgment-based, continuous, and multicomponent interventions. It links quantitative delivery patterns to qualitative explanations and makes previously implicit operational decisions visible. The integrated tables and **Supplemental Digital Content 1** (https://links.lww.com/CCX/B697) provide reusable starting points.

This synthesis has several limitations. It was retrospective, was not governed by a prospectively registered cross-trial protocol, and included four trials selected because they had applied or informed POETIC and had sufficiently detailed completed process evaluations. Because of this selection, the synthesis may overrepresent applications that fit the framework well. The data sources, timing, measurement, and analytic methods also differed across trials, and we did not reanalyze the primary datasets. Historical missing-data rules for combined scores were not consistently reported.

L.E. and closely linked research teams contributed to several applications and to the development of POETIC. This familiarity supported continuity but may also have encouraged confirmation of the framework’s existing assumptions. Cross-checking, team discussion, attention to contradictory findings, and explicit limits on applicability reduced but could not remove this risk.

All four trials were conducted in U.K. ICUs. The guidance therefore requires testing in other health systems, resource settings, and regulatory environments. Site-level scores may be unstable when few participants or eligible occasions contribute, and trial-specific QoD scores and categories should not be assumed to have established measurement properties or external validity. Prospective monitoring may itself alter behavior. The toolkit and governance safeguards are practical guidance derived from these applications, not prospectively validated methodological standards. Future work should evaluate their feasibility, acceptability, and usefulness in trials applying POETIC from the design stage.

## CONCLUSIONS

Applying POETIC across four critical care trials allowed us to translate its conceptual structure into a practical toolkit. Trials need not measure every dimension or calculate QoD. This requires measures matched to the intervention mechanism: longitudinal Context, meaningful Fidelity, Dose, and Reach measures where applicable, optional transparent QoD, clear mixed-methods integration, and governance proportionate to how findings will be used. This approach can distinguish intervention effects from delivery limitations while protecting trial integrity.

## ACKNOWLEDGMENTS

We gratefully acknowledge funding from the Medical Research Council (MRC), which supported this work in part through a four-year doctoral fellowship as part of the All-Ireland Hub of the MRC Network of Hubs for Trials Methodology Research.

We also acknowledge the trial teams and participants involved in Biomarker-guided antibiotic stewardship in suspected ventilator-associated pneumonia (VAPrapid-2), Provision of Psychological Support to People in Intensive Care (POPPI), Sedation AND Weaning In CHildren (SANDWICH), and Alpha-2 agonists for sedation to produce Better outcomes from critical illness (A2B).

## Supplementary Material
