# Supplementary material for: From Framework to Toolkit: Practical Guidance for Using the PrOcess Evaluation of Trials In Critical care (POETIC) Framework
Source: Crit Care Explor. 2026 Oct 27;8(11):e1503. doi: 10.1097/CCE.0000000000001503 (PMC13716417; doi:10.1097/CCE.0000000000001503)
Supplement: Supplementary file 1 [file cc9-8-e1503-s001.pdf]

POETIC practical toolkit: planning, measurement, governance, and reporting worksheets

Companion to: *From framework to toolkit: practical guidance for using the PrOcess Evaluation of Trials In Critical care (POETIC) framework*

This supplemental digital content accompanies the retrospective comparative synthesis and contains an applicability guide (Supplemental Table 1) and five worksheets following the five steps in the POETIC toolkit. Use these as adaptable starting points: they do not prescribe universal thresholds or require a Quality of Delivery (QoD) score. Any QoD thresholds or categories should be defined within the individual trial and justified by the intervention mechanism, intended interpretation, and appropriate empirical or theoretical evidence. Measures, thresholds, recipients, review times, permitted actions, and safeguards should be agreed before access to comparative outcome data and aligned with the intervention logic model, protocol, process evaluation analysis plan, and relevant oversight documents.

Tool 1. Define the intervention and choose useful measures

A. Intended use

Decision	Trial-specific entry
Primary interpretive question<br>*(What do you need the process evaluation to explain?)*	
Intended mode	☐ End-of-trial explanation ☐ Prospective monitoring ☐ Governed during-trial interpretive use
Unit(s) of analysis	☐ Patient ☐ Deliverer ☐ Site ☐ Time period ☐ Other:
Core intervention functions<br>*(What must the intervention achieve?)*	
Core components	
Adaptable components and permitted adaptations<br>*(What may change without undermining how the intervention is expected to work?)*	
Will relevant process evaluation analyses be completed before access to comparative outcome data?	☐ Yes ☐ No; explain:

B. Dimension specification

Dimension	Question	Measure/source	Denominator/eligible opportunity	Timing	Planned analysis
Context	What may shape delivery here and now?				
Fidelity	Were core functions/components delivered as intended?				
Dose	How much was delivered and received?				
Reach - patients	Which eligible patients were exposed/engaged?				
Reach - staff/sites	Which intended staff or sites were reached or prepared to deliver the intervention?				
Trial processes (record separately from POETIC intervention delivery dimensions)	What affected recruitment, retention, or data collection?				
QoD, if justified	Does integration add information beyond components?				

C. Applicability check

Supplemental Table 1. Applicability guide for selecting POETIC measures by intervention type

Intervention type	Emphasize	Dose/QoD considerations	Example
Protocolized, repeatable components	Component Fidelity, Dose measured only on eligible occasions, patient/staff Reach, Context	QoD is often feasible if core components and opportunities are prespecified	SANDWICH
Continuous therapy	Treatment Fidelity, exposure over time, assessment frequency, eligible-patient Reach	Define clinically coherent time windows; a composite may combine treatment, target, assessment, and Reach	A2B
Multicomponent program	Core-function Fidelity, component Dose, deliverer and recipient Reach	Weighting may be justified when components differ in importance to how the intervention is expected to work; report each component alongside the combined score.	POPPI
Diagnostic or judgment-based decision support	Availability and decision-concordance Fidelity, eligible-exposure Reach, attitudes and risk perceptions	Dose may add little information or repeat what Fidelity already shows; avoid a combined score that suggests repeated exposure when the intervention is one-off.	VAPrapid-2
One-off procedure	Procedural Fidelity and eligible-patient Reach	Dose may duplicate Fidelity; report components rather than forcing QoD	Single procedural intervention
Tailored or adaptive intervention	Fidelity to core functions, documentation and justification of adaptation/tailoring, Reach	Assess permissible adaptation separately from non-adherence; simple compliance scores may mislead	Clinician-tailored care pathway

- Is Dose distinct from Fidelity for this intervention? ☐ Yes ☐ No ☐ Uncertain
- Can patient, staff, and site Reach be distinguished from trial processes such as recruitment, retention, and data collection? ☐ Yes ☐ No
- Are eligible opportunities observable? ☐ Yes ☐ No
- Can clinically justified exceptions be separated from non-adherence? ☐ Yes ☐ No
- Would a composite hide a clinically important component profile? ☐ Yes ☐ No
- If any answer creates ambiguity, report components separately and use qualitative explanation before considering QoD.

Tool 2. Track Context over time

Complete at baseline, at prespecified intervals, after trigger events, and at a defined final assessment point. Define this point in the protocol; for example, the end of intervention delivery at a site or the end of the final delivery phase. State whether it will occur before comparative outcome data are available. Record both the current state and the change since the previous assessment.

Date/window	Site	Domain	Minimum observation/measure	Change since last assessment	Plausible effect on Fidelity/Dose/Reach	Evidence source	Follow-up needed
		Unit Culture	Protocol norms; learning/psychological safety; multidisciplinary working; tolerance of uncertainty			Interview/survey/observation	
		Organizational Structure	Unit type/size; decision authority; role clarity; handover and multidisciplinary routines			Unit profile/interview	
		Resources	Staffing, skill mix, turnover, workload/surge, research support, equipment/supply			Routine metrics/event log	
		Usual Practice	Baseline care, distance from intervention, competing protocols/trials, practice drift			Audit/routine data/interview	
		Attitudes and Perceptions	Acceptability, confidence, risk/safety concern, equipoise, burden, legitimacy			Interview/survey	
		External/temporal events	Guideline, outbreak, reconfiguration, workforce disruption, supply interruption			Dated event log	

Suggested triggers for unscheduled assessment

- sustained change in delivery or recruitment;
- major staffing change or loss of research support;
- Intensive care unit (ICU) surge, outbreak, or service reconfiguration;
- change to relevant national/local guidance;
- new external evidence likely to affect equipoise or usual practice;
- unexpected missing component data or a problem with how the data are defined; or
- safety or integrity concern requiring escalation through existing pathways.

Tool 3. Build QoD only when it adds value

A. Component specification

Component	POETIC dimension	Mechanism/function represented	Numerator	Denominator	Threshold rationale	Scale	Weight	Minimum data required
1					☐ Intervention mechanism<br>☐ Clinical/guideline threshold<br>☐ External evidence<br>☐ Observed distribution<br>☐ Expert/team consensus			
2					☐ Intervention mechanism<br>☐ Clinical/guideline threshold<br>☐ External evidence<br>☐ Observed distribution<br>☐ Expert/team consensus			
3					☐ Intervention mechanism<br>☐ Clinical/guideline threshold<br>☐ External evidence<br>☐ Observed distribution<br>☐ Expert/team consensus			
4					☐ Intervention mechanism<br>☐ Clinical/guideline threshold<br>☐ External evidence<br>☐ Observed distribution<br>☐ Expert/team consensus			

B. Aggregation and analysis decisions

Decision	Prespecified rule
Equal or differential weighting and rationale<br>*(Will components count equally? If not, why?)*	
Patient-to-site aggregation<br>*(How will patient results become one site result?)*	
Time-window aggregation<br>*(How will results from different periods be combined?)*	
Continuous score range<br>*(What are the lowest and highest possible scores?)*	
Categories and rationale<br>*(Which scores mean low, moderate, or high—and why?)*	
Required components<br>*(Which measures must be present?)*	
Missing component rule<br>*(What happens if an essential measure is missing?)*	☐ Not classifiable ☐ Prespecified rescaling ☐ Prespecified imputation; method:
Small denominator rule<br>*(What happens when very few patients or eligible occasions are available?)*	
Sensitivity analyses<br>*(Would other reasonable scoring choices change the conclusion?)*	
Planned relation to trial outcomes<br>*(How will QoD be compared with trial outcomes?)*	
How component values will be reported beside the composite<br>*(How will readers see what drove the overall score?)*	

C. Worked example using the published A2B scoring method

The example values below are hypothetical; the indicators, cut points, weighting, and observed category labels reproduce the published A2B process evaluation method (see main manuscript reference 15).

**A2B indicator for a hypothetical site**	**Hypothetical site value**	**Published 0–3 scoring rule**	**Score**
Correct randomized treatment every day during first 4 days (Fidelity)	85%	≥80% = 3	3
Richmond Agitation-Sedation Scale (RASS) -2 or lighter at any point during first 4 days (Fidelity)	73%	60-79% = 2	2
RASS assessed at least daily during first 4 days (Dose)	92%	≥80% = 3	3
Eligible patients proceeding to randomization (Reach)	55%	40-59% = 1	1
**Total, equal weights**		**0–12 possible**	**9**

These thresholds and categories reproduce those used for the observed A2B data and are trial-specific descriptive constructs provided only for illustration. Their measurement properties and external validity have not been established, and they should not be transferred to another intervention or trial without independent justification. In this hypothetical example, a total of 9 indicates moderate adherence (low 6-7; moderate 8-9; high 10-12). Component reporting remains essential: the total shows that the principal limitation is Reach, not treatment Fidelity or assessment Dose.

For future applications, we recommend reporting a required component as missing and treating the composite as not classifiable unless a different rule was justified and specified in advance, such as rescaling the score or imputing (filling in) the missing value. The minimum amount of contributing data should also be specified.

Tool 4. Set rules for use during the trial

A. Roles, access, and reporting intervals

Item	Prespecified entry
Process evaluation lead and team	
Staff permitted to view site-level delivery data	
Access to comparative outcome data and separation of roles	
Recipients of aggregate or blinded summaries	
Trial-management, steering, and data-monitoring responsibilities	
Reporting interval	☐ Monthly ☐ Quarterly ☐ Phase-based ☐ Triggered ☐ Other:
Report format and version control	
Decision-log custodian(s)	
Safety/data-integrity escalation pathway	

B. Action boundary

Action	Status	Conditions/authority
Resolve missing fields or data-definition errors	☐ Permitted ☐ Restricted ☐ Prohibited	
Trigger prespecified explanatory sampling	☐ Permitted ☐ Restricted ☐ Prohibited	
Provide aggregate/blinded Context and delivery summary	☐ Permitted ☐ Restricted ☐ Prohibited	
Give site-specific performance feedback	☐ Permitted ☐ Restricted ☐ Prohibited	
Prompt additional intervention training	☐ Permitted ☐ Restricted ☐ Prohibited	
Change indicators, thresholds, or weights	☐ Permitted ☐ Restricted ☐ Prohibited	
Modify intervention content or dose	☐ Permitted ☐ Restricted ☐ Prohibited	
Access comparative outcome data	☐ Permitted ☐ Restricted ☐ Prohibited	

Permitted = allowed; restricted = allowed only under stated conditions; prohibited = not allowed. Status should be determined in accordance with the trial protocol, statistical analysis plan, and relevant oversight arrangements; this worksheet does not itself authorize an action. If feedback is intended to improve delivery, describe it as an intervention component, apply it consistently according to prespecified rules, record who receives it, and consider how it could affect the planned treatment effect and interpretation of the trial findings.

C. Decision log

Date	Information reviewed	Recipients	Interpretation	Action authorized	Authority/rationale	Potential bias/contamination considered	Follow-up

Tool 5. Combine and report the evidence

A. Site-by-time joint display

Site/time window	Context	Fidelity	Dose	Reach	QoD, if used	Qualitative explanation	Integration judgment	Alternative explanation/data gap
							☐ Agreement<br>☐ Complementarity<br>☐ Disagreement	

B. Reporting checklist

- Restate the intervention logic model and distinguish core from adaptable components.
- Define each POETIC dimension used and explain why any dimension was not measured or was applied differently.
- Identify the unit or units of analysis and define denominators using the patients or delivery opportunities that were actually eligible.
- Report baseline Context, changes over time, between-site variation, and trigger events.
- Describe qualitative sampling, data collection, the chosen analytic approach, independent checking, and how findings that did not fit the main interpretation were handled.
- Explain how qualitative and quantitative findings were integrated.
- For QoD, report every component, threshold, rationale, weight, range, aggregation, category, and missing-data rule.
- Display component results beside any composite and identify components based on very few patients or eligible delivery opportunities.
- State whether process evaluation personnel had access to comparative outcome data and, if not, how separation was maintained.
- State who received outputs, when, in what form, and what actions were permitted.
- Disclose any implementation material, feedback, training, or trial-conduct action informed by process evaluation findings.
- Describe safeguards against performance bias, contamination between randomized groups, and unintended changes in intervention delivery.
- Report the limits of transferability and how investigator overlap or prior involvement may have influenced interpretation.
